# Cytotoxic response of tumor-infiltrating lymphocytes of head and neck cancer slice cultures under mitochondrial dysfunction

**DOI:** 10.3389/fonc.2024.1364577

**Published:** 2024-03-07

**Authors:** Maria do Carmo Greier, Annette Runge, Jozsef Dudas, Roland Hartl, Matthias Santer, Daniel Dejaco, Teresa Bernadette Steinbichler, Julia Federspiel, Christof Seifarth, Marko Konschake, Susanne Sprung, Sieghart Sopper, Avneet Randhawa, Melissa Mayr, Benedikt Gabriel Hofauer, Herbert Riechelmann

**Affiliations:** ^1^ Department of Otorhinolaryngology, Head and Neck Surgery, Medical University of Innsbruck, Innsbruck, Austria; ^2^ Institute for Clinical and Functional Anatomy, Medical University Innsbruck (MUI), Innsbruck, Austria; ^3^ INNPATH GmbH, Institute for Pathology, Innsbruck, Austria; ^4^ Clinic for Internal Medicine V, Medical University Innsbruck, Innsbruck, Austria; ^5^ Department of Otolaryngology, Rutgers University Medical School, Newark, NJ, United States; ^6^ ViraTherapeutics GmbH, Rum, Austria

**Keywords:** immune response, cytotoxic T-cells, mitochondrial electron transport chain, head and neck carcinoma, mitochondrial dysfunction

## Abstract

**Background:**

Head and neck squamous cell carcinomas (HNSCC) are highly heterogeneous tumors. In the harsh tumor microenvironment (TME), metabolic reprogramming and mitochondrial dysfunction may lead to immunosuppressive phenotypes. Aerobic glycolysis is needed for the activation of cytotoxic T-cells and the absence of glucose may hamper the full effector functions of cytotoxic T-cells. To test the effect of mitochondrial dysfunction on cytotoxic T cell function, slice cultures (SC) of HNSCC cancer were cultivated under different metabolic conditions.

**Methods:**

Tumor samples from 21 patients with HNSCC were collected, from which, SC were established and cultivated under six different conditions. These conditions included high glucose, T cell stimulation, and temporarily induced mitochondrial dysfunction (MitoDys) using FCCP and oligomycin A with or without additional T cell stimulation, high glucose and finally, a control medium. Over three days of cultivation, sequential T cell stimulation and MitoDys treatments were performed. Supernatant was collected, and SC were fixed and embedded. Granzyme B was measured in the supernatant and in the SC via immunohistochemistry (IHC). Staining of PD1, CD8/Ki67, and cleaved­caspase­3 (CC3) were performed in SC.

**Results:**

Hematoxylin eosin stains showed that overall SC quality remained stable over 3 days of cultivation. T cell stimulation, both alone and combined with MitoDys, led to significantly increased granzyme levels in SC and in supernatant. Apoptosis following T cell stimulation was observed in tumor and stroma. Mitochondrial dysfunction alone increased apoptosis in tumor cell aggregates. High glucose concentration alone had no impact on T cell activity and apoptosis. Apoptosis rates were significantly lower under conditions with high glucose and MitoDys (p=0.03).

**Conclusion:**

Stimulation of tumor-infiltrating lymphocytes in SC was feasible, which led to increased apoptosis in tumor cells. Induced mitochondrial dysfunction did not play a significant role in the activation and function of TILs in SC of HNSCC. Moreover, high glucose concentration did not promote cytotoxic T cell activity in HNSCC SC.

## 1 Introduction

### 1.1 HNSCC tumor microenvironment

Head and neck squamous cell carcinomas (HNSCC) are common malignancies with an unfavorable prognosis and severe disease burden. They develop from mucosal epithelial cells, especially in the oral cavity, pharynx, larynx and sinunasal tract. These tumors exhibit great intra- and inter-individual heterogeneity and develop in a complex and hostile tumor microenvironment (TME). Within the microarchitecture of HNSCC, tumor cells often form aggregates of different sizes, sometimes referred to as tumor cell nests. These tumor cell aggregates lie within a tumor stroma of extracellular matrix that harbors a heterogeneous mixture of stromal cells, including endothelial cells, cancer-associated fibroblasts (CAF), and immune cells, of which tumor infiltrating lymphocytes (TILs) are the major type (1–3).

### 1.2 Immune evasion in HNSCC

In order to survive in the TME, the tumor cells must evade immune surveillance. The microarchitecture of the TME is an important factor in achieving this. In the common immune exclusion phenotype, the immune cells are spatially restricted to the stroma and hardly penetrate the tumor cell aggregates (3, 4). The barrier between tumor cell aggregates and stroma is due to the surrounding thick ECM, the absence of lymphatic vessels, and a disorganized vasculature. Hypoxic conditions, hyperacidity, and substrate deficiency within the tumor cell aggregates also prevent immune cell infiltration (5, 6). Another essential escape mechanism is the immune checkpoint programmed cell death protein 1 (PD-1) (7). PD-1 is mainly expressed on T cells, B cells and natural killer (NK) cells. It inhibits the activity of these cells when activated by its ligand PD-L1, which is frequently expressed on HNSCC tumor cells (8). Interruption of PD-1/PD-L1-mediated immune evasion by monoclonal antibodies is the basis of modern immune checkpoint inhibitors (ICI) and represents a major therapeutic breakthrough, particularly in metastatic and recurrent HNSCC (9). Persistent PD-1 expression has also been recognized as a marker of T cell exhaustion (10, 11), which is another mechanism by which HNSCC tumor cells escape immune surveillance (12). Due to metabolic stress in the microenvironment combined with sustained TCR-mediated stimulation, effector T lymphocytes undergo mitochondrial dysfunction (MitoDys) and, similar to tumor cells, switch from OXPHOS to aerobic glycolysis (13, 14). This leads to competition between T cells and tumor cells for nutrients, especially glucose, and impaired T cell effector functions (15, 16). In HNSCC, MitoDys can be induced experimentally with FCCP (carbonyl cyanide-4-(trifluoromethoxy) phenylhydrazone) and oligomycin A (17).

### 1.3 HNSCC slice cultures

Although cell cultures are excellent models for mechanistic studies of the metabolism and cellular function of tumor cells and immune cells, they do not adequately reflect the complex three-dimensional relationships in the TME: the different cell populations, the spatial compartmentalization in tumor and stroma, and the high intra- and inter-individual heterogeneity of HNSCC. In recent years, various *in vitro* and ex vivo models have been developed that allow a more realistic investigation of the events in the TME (18). The cultivation of tissue sections from tumor biopsies of patients (slice cultures) reflects the complex relationships in the TME more effectively than cell cultures do (19, 20). However, experimenting with HNSCC slice cultures (SC) requires access to a clinical facility where fresh tumor tissue samples from patients with HNSCC are available. Compared to cell cultures, the possible experimental interventions are limited. Although SC of HNSCC remain viable, maintain their microenvironment, and respond to experimental immunologic interventions (21, 22), they can only do so for a few days. Moreover, they are only available in limited quantities and complex procedures like transgenic interventions are hardly feasible. While cell cultures can be accurately characterized by flow cytometry, the cell separation required for flow cytometry of SC may lead to unreliable results due to possible epitope loss. For this reason, histological evaluation methods are primarily used to analyze SC.

### 1.4 Image cytometry

Modern image cytometric methods overcome some of the limitations of conventional histopathological analysis. These methods involve the quantitative evaluation of chromogen or fluorescence intensities, based on the segmentation of single cells in tissue sections. This approach yields multiparametric information including size, compactness, and location of cells with chromogenic or fluorescence intensity for each biomarker (23). With the simultaneous use of differently labeled antibodies, multiplex cytometry is possible, allowing a differentiated analysis of the phenotype, spatial distribution, and activation of cells (24) in the TME. Recent imaging cytometric techniques allow automatic differentiation between tumor cell aggregates and tumor stroma in HNSCC (25).

### 1.5 Study aims

Using SC from patients with HNSCC, we investigated the effects of MitoDys on TME. Specifically, we investigated how MitoDys affects apoptosis in tumor cell aggregates and tumor stroma, how glucose concentration modulates MitoDys-induced apoptosis, and how T cell activation affects apoptosis in tumor and stroma of HNSCC slice cultures. In particular, we were interested in whether MitoDys interferes with the effects of T cell activation. In addition, we examined PD-1 expression under these experimental conditions as a possible indicator of T cell exhaustion.

## 2 Methods

### 2.1 Study population and preparation of HNSCC slice cultures

All patients with incident, locally advanced HNSCC treated at the Department of Otorhinolaryngology - Head and Neck Surgery of the Medical University of Innsbruck between January 2022 and September 2022 who agreed to participate were consecutively included. Inclusion criteria were patient age over 18 years, who underwent diagnostic endoscopy under anesthesia (26) with tumor biopsy and had a locally advanced primary tumor (T3-T4). Patients were excluded if there was a contraindication to endoscopy under anesthesia or if the histopathology was not HNSCC. This study was approved by the Ethics Committee of the Medical University of Innsbruck (EC number: 1199/2019). Written informed consent was obtained from all patients. Tumor biopsies from 21 patients with newly diagnosed histologically confirmed HNSCC were obtained. Seventeen primary tumors were located in the oropharynx, three in the oral cavity, and one in the larynx. Six tumors were p16 positive. The patients were between 31 and 87 years old (average 61.4 years), four patients were female. All patients but one had UICC stage III or IV HNSCC by clinical and radiologic evaluation. The tissue samples were taken with biopsy forceps from a non-necrotic tumor area during diagnostic endoscopy under anesthesia and immediately brought to the laboratory for the preparation of slice cultures (SC). Six slices with a thickness of 250 μm were cut from each patient sample using the Compresstome® VF-310-0Z (Precisionary Instruments LLC, MA, USA) and then plated (21).

### 2.2 Culture conditions

SC were submerged in a 24-well plate (Corning Incorporated-Life Sciences, Durham, USA) under six different conditions (**Table 1**). One ml of serum-free keratinocyte medium (Keratinocyte SFM; #10724-011, Gibco, Grand Island, NY, USA) was added to all six wells. Keratinocyte SFM is a complete serum-free medium supplemented with human recombinant epidermal growth factor and bovine pituitary extract. It was supplemented with Gibco Antibiotic-Antimycotic (#15240062, Thermo Fischer Scientific, Rochester, NY, USA) diluted 1:100 in the medium, resulting in a final concentration of 100 µg/mL streptomycin, 250 ng/mL amphotericin B and 100 units/mL penicillin.

**Table 1:** SC cultivation conditions, glucose concentrations and compounds used for mitochondrial dysfunction and T cell stimulation.

Condition No.	Shortcut	Glucose conc.	Mitochondrial Dysfunction	T Cell stimulation
1	Control	5.8 mmol/l		
2	MitoDys	5.8mmol/l	FFCP/oligomycin	
3	T cell activation	5.8mmol/l		T cell activation kit
4	HighGluc	25 mmol/l		
5	HighGluc+MitoDys	25 mmol/l	FFCP/oligomycin	
6	MitoDys+T cell Stim	5.8mmol/l	FFCP/oligomycin	T cell activation kit

**Figure 1:**
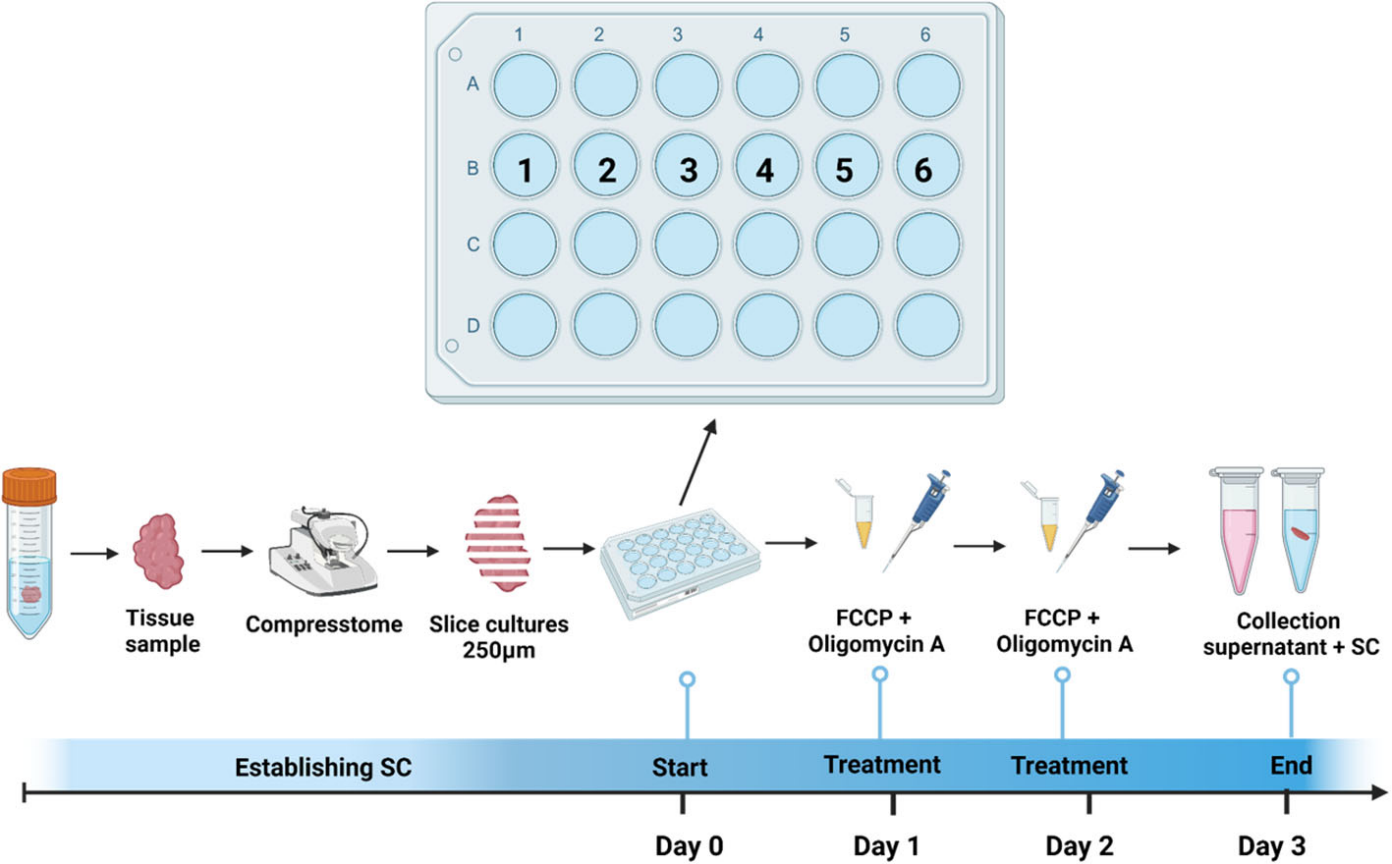
Treatment and cultivation procedure. SC were obtained from tumor samples of patients with incident head & neck squamous cell carcinoma during diagnostic endoscopy and sliced using Compresstome. Six SC per patient were plated in a 24-well plate (1=Control; 2=MitoDys; 3= T cell stim; 4= HighGluc; 5=HighGluc+ MitoDys and 6=MitoDys +T cell stim; see **Table 1**). After one day, three wells (2,5,6) were treated with FCCP + oligomycin A for 50min. After another day, this procedure was repeated, after 3 days in total, SC were fixed, and supernatant was collected.

To experimentally induce MitoDys (Conditions 2, 4 and 6; **Table 1**), 1µl of a 1mM mixture of FCCP (carbonyl cyanide-4-(trifluoromethoxy) phenylhydrazone, #370-86-5, Sigma Aldrich, Darmstadt, Germany) and oligomycin A (#75351-5MG, Sigma Aldrich, Darmstadt, Germany) was added to the wells after 24 hours for 50 minutes, according to treatment conditions described in the XFp Seahorse Analyzer protocol (17). This procedure was repeated after a further 24 hours (**Figure 1**). FCCP uncouples the OXPHOS and oligomycin A blocks ATP synthase. Together, they block mitochondrial ATP synthesis.

For T cell stimulation (T Cell stim; conditions 3 and 4; **Table 1**), 30 µl of a T Cell Activation/Expansion Kit (#130-091-441, Miltenyi Biotec, Gladbach, Germany) was added to the wells. The kit consists of anti-biotin-MACSiBead particles and biotinylated antibodies against human CD2, CD3 and CD28. The anti-biotin-MACSiBead particles loaded with the biotinylated antibodies are used to simulate antigen-presenting cells. To achieve T cell expansion beyond T cell activation, the addition of rIL-2 is required on day 5 and 14 of the culture (Miltenyi Biotec, #130-091-441 data sheet), which was not done here.

For high glucose concentrations (HighGluc; conditions 5 and 6; **Table 1**), Keratinocyte SFM was augmented to 25mM with 45% glucose solution (#25-027-CI, Corning, Arizona, USA).

### 2.3 Collection of supernatant, ELISA, and dot blot

After 72 hours, the SC were fixed, and the supernatants were collected from each well. (**Figure 1**) The pH was measured (InoLab ph level 1, Inolab - wtw, Weilheim, Germany). Lactate concentration was detected using the Lactate Assay Kit II (#KA0834, Abnova, Taipei, Taiwan) and measured using an ELISA plate reader (Anthos 2010, Salzburg, Austria). Granzyme B was measured using dot blot analysis. Circles were drawn with a pencil on the Amersham Protran 0.2 µm Nitrocellulose Blotting Membrane (#10600001, GE Healthcare Life Sciences, Amersham, Buckinghamshire, UK) for each supernatant sample. The membranes were briefly treated with methanol and then dried. From each supernatant sample, 20 µl was pipetted into a circle on the membrane. After drying, the membranes were rehydrated in TBS and blocked for one hour at room temperature in Invitrogen TBS Starting Blocking Solution (#37542). The membranes were then incubated with the primary granzyme B antibody IgG2b 1:200 (#3002-MSM4-P1, Invitrogen, Darmstadt, Germany) in Invitrogen TBS Starting Blocking Solution with 0.2% Tween 20 overnight. Anti-mouse IgG IR 800 1:10000 (Azure Biosystem, Houston, TX, USA) was used to detect the antibody reaction. The optical densities of the drawn circles in the membranes were measured with ImageJ 1.46r (1.6.0_20, National Institutes of Health, USA), and the background densities were subtracted.

### 2.4 SC immunostaining procedures

After cultivation period of 72h, SCs were fixed in 4% paraformaldehyde (#FN-10000-4-1, SAV Liquid Production GMBH, Flintsbach am Inn, Germany) overnight (4° C) and washed with phosphate-buffered saline (PBS; Fresenius Kabi GmbH, Bad Homburg v.d.H, Germany) the next day. Fixed SC were prepared for paraffin embedding with the Histos 5 microwave system (Milestone, Bergamo, Italy) and 5 µm microtome sections were prepared afterwards [2] and dewaxed [35]. Hematoxylin-Eosin (HE) staining followed the manufacturers´ protocol (#1.05174.0500, Merck KGaA, Darmstadt, Germany).

Following the protocol of Fischer et al. (27), cleaved caspase-3 (CC3) staining was performed using the fully automated immunostaining system Ventana Discovery Ultra immunostainer (Ventana Roche Discovery Classic, Tucson, AZ, USA) and the CC3 antibody (1:400x, polyclonal rabbit, #9661, Cell Signaling Technology, Danvers, MA, USA).

For granzyme B detection mouse monoclonal IgG2b antibody 1:200 (#3002-MSM4-P1, Invitrogen, Darmstadt, Germany) was used and for PD1 staining the EH33 antibody 1:100 (mouse monoclonal, Mob573) from Diagnostic Biosystems (Baltimore, MD, USA). For detection of primary mouse and rabbit antibodies, Ventana universal secondary antibody (#05268877) was used. The reaction was fully developed using Ventana DAB Map Detection Kit (#05266360001, Roche Diagnostics, Mannheim, Germany). Cell nuclei were counterstained using Hematoxylin (#5277965001, Roche Diagnostics). After staining procedure, the slides were dehydrated, mounted with Entellan (MERCK, Darmstadt, Germany) and dried overnight. T-cell proliferation was characterized by immune fluorescence staining with CD8 mouse monoclonal IgG2b 1:50 (#NCL-L-CD8-4B11 Novocastra, Manchester, UK), combined with prediluted mouse monoclonal IgG1 Ki67 antibody (E059, Linaris, Dossenheim, Germany). Mouse IgG1 was detected by Alexa 488 conjugated anti-mouse secondary antibody; mouse IgG2b was detected by Alexa 555 conjugated secondary antibody (Invitrogen, Darmstadt, Germany). Nuclei were counterstained with 4′,6-diamidin-2-phenylindolethen (DAPI, 1:46.000, Thermo Fisher Scientific, Darmstadt, Germany). Autofluorescence was reduced with the Vector TrueVIEW Auto fluorescence Quenching Kit (#VEC-SP-8400, Vector Laboratories, Burlingame, California, USA) [35]. After staining procedures, the slides were mounted with Vectashield Vibrance (Vector Laboratories) [2]. PD1, CD8 and Ki67 clone MIB-1 are diagnostic antibodies which are continuously validated by the provider. Granzyme B was validated in human tonsils as suggested by the provider.

### 2.5 Image cytometry

For immunofluorescence image acquisition, TissueFAXS PLUS (TissueGnostics GmbH, Vienna, Austria) was used. Images were acquired with Zeiss Axio Imager Z2 Microscope (Jena, Germany) and apochromat 40x, 0.6 air lens. As fluorescence light source Zeiss HBO 100 Mercury Lamp 42301101 (Jena, Germany) was used. For excitation and detection, the following filters were used: Zeiss filter set 44 for Ki67 (AF488), filter set 20 for CD8 (AF555) and filter set 49 for DAPI (24). Colors were arbitrarily chosen for each channel: green for Ki67, red for CD8 and blue for DAPI. For preview and acquisition PCO pixelfly CCD camera (PCO AG, Kelheim, Germany) was used.

**Figure 2:**
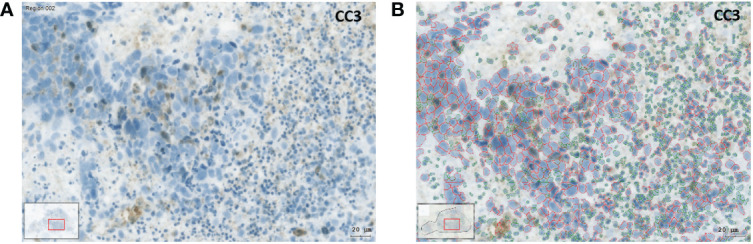
Image analysis with the HistoQuest software using the example of an IHC preparation with cleaved caspase 3 (CC3). **(A)** SC after 3 days in culture under control conditions. The brown CC3 reaction products can be recognized. **(B)** Identification of tumor and stromal cells using the HistoQuest software from the same sample. The cells circled in red were identified by the software as tumor cells, the cells circled in green as inflammatory cells (bar corresponds to 20µm).

TissueFAXS PLUS, containing microscope and lens, as described above, was also used for enzyme immunohistochemistry. For image acquisition, PIXELINK camera (PIXELINK, Ottawa, Canada) was used. Relative staining intensities were determined in whole SC using HistoQuest (TissueGnostics GmbH, Vienna, Austria). Acquired images were imported in HistoQuest. Regions which were not possible to analyze (necrotic, too small) were excluded by the users. Using the possibilities of HistoQuest, the engine single reference shade was used after defining the main colors of Hematoxylin counterstain (blue) and DAB-reaction (brown). The next step was the optimization of cell nuclei recognition, using previous published knowledge (24). The following procedures served the recognition of immunohistochemical staining, considering its localization, membranous or cytoplasmic. Tissue was stratified into macrostructures, to differentiate between stroma and tumor cells. Tumor and stroma cells were identified based on their different hematoxylin equivalent diameter and hematoxylin area (**Supplementary Figure 1**). Cells were recognized by hematoxylin counterstaining and the immunohistochemical signals were attributed to the identified cell nuclei (**Figure 2**). Staining intensities were provided by the software based on intensity values for all pixels grouped to cells, which were identified by their cell nuclei (**Supplementary Tables 1**-**6**). Mean intensity is a dimensionless number representing the intensity in the color of the IHC reaction products of all pixels grouped into tumor and stroma categories, as described above (28). HistoQuest provides various data including total cell count, specimen area, mean color intensity of IHC reaction products, and mean fluorescence intensities. Pathologist (Su.S.) and histologist (M.K.) supervised morphological procedures.

### 2.6 Data analysis

The parameters investigated followed a gamma distribution, with the individual test conditions clustered within the patients. Accordingly, a generalized estimating equations model was chosen for the evaluation, based on the gamma distribution with a logarithmic link function including a constant term. The parameters were estimated using the maximum likelihood method and the p-values were calculated according to Wald. The alpha error adjustment was carried out using the least significant difference method. The mean values predicted by the model (estimated marginal means) and their 95% confidence intervals (95% CI) were reported as outcomes. Comparisons of parameters across all experimental conditions were analyzed using the Mann-Whitney U test, with the medians and the 25th and 75th percentiles reported. Calculations were performed with SPPS Statistics Ver. 27 (IBM, Armonk, NY) and graphically presented using GraphPad Prism 9 (GraphPad Software, San Diego, CA, USA).

## 3 Results

### 3.1 SC in HE stains at day 0 and 3

**Figure 3:**
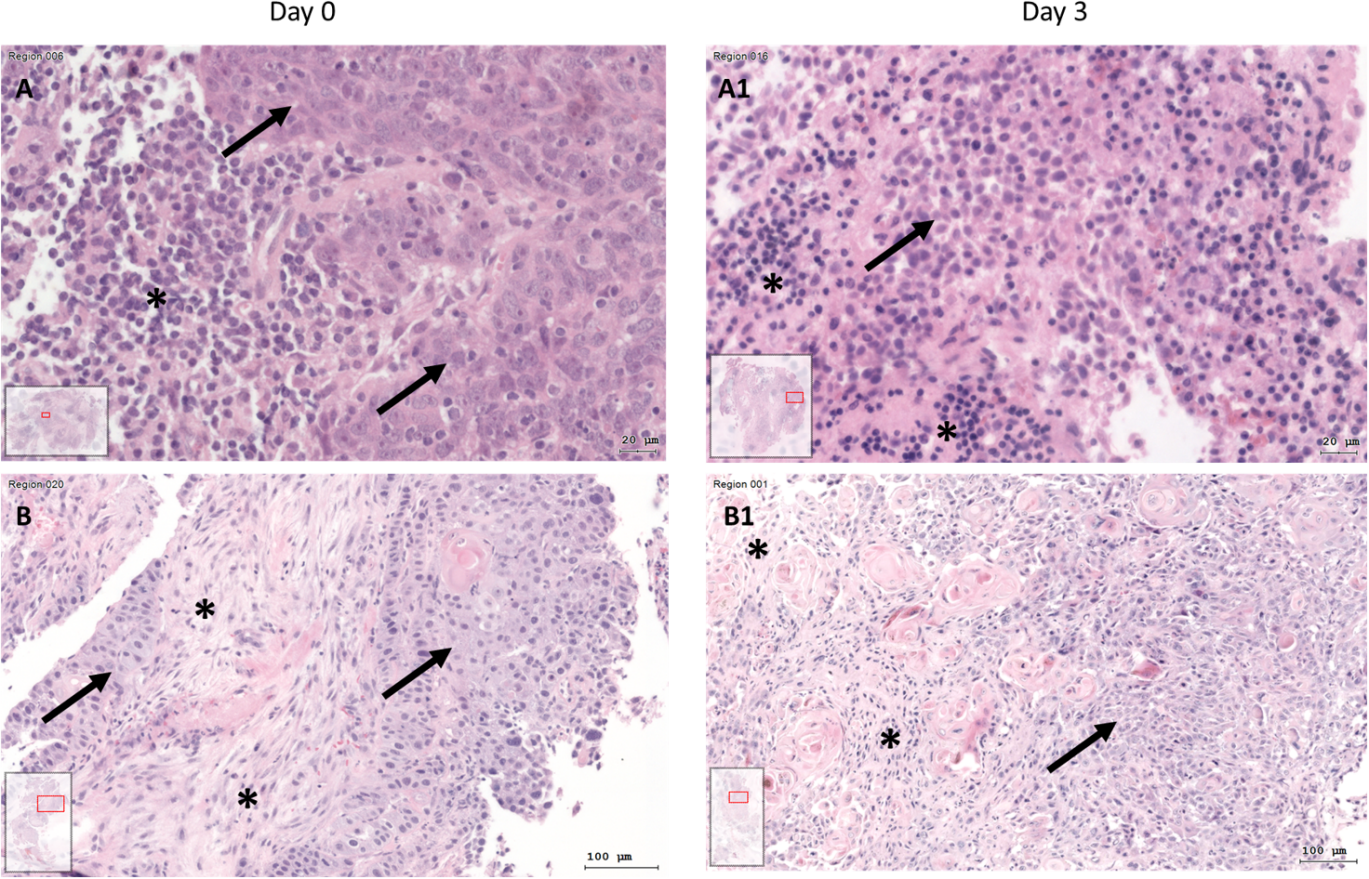
HE staining of tissue at day 0 (A+B) and SC after day 3 (A1+B1) of two different patients. **(A)** HNSCC of Oropharynx in a male patient aged 75 years at day 0 (A) and day 3 (A1; Bar: 20µm; arrow: tumor cells; asterisk: inflammatory cells, inlets: position of the image section in the whole slide). At day 0, there are visible tumor cells (arrow) and at day 3 there are tumor cells (arrow) and inflammatory cells (asterisk). B) HE staining of HNSCC of oral cavity (male, 37 years old) at day 0 **(B)** and day 3 (B1; bar: 100µm). Tumor cells (arrows) and tumor stroma (asterisk) are present at day 0 and day 3 of cultivation.

After 3 days of cultivation, the SC were fixed, embedded, and sliced. Compared to day 0, HE stains showed preserved tissue architecture with preserved cells. Tumor cell aggregates and tumor stroma could also be differentiated on day 3 (**Figure 3**). However, across all test conditions, the cell density was lower on day 3 (median 9089; quartiles 4530 to 17478 cells/mm²) than on day 0 (median 14785; quartiles 9350 to 23527 cells/mm²; Wilcoxon p<0.001).

### 3.2 Cleaved caspase 3 (CC3)

Caspase-3 is a canonical actor of apoptosis. The activation of caspase-3 requires the proteolytic cleavage of its inactive zymogen into activated p17 and p12 fragments. Cleaved Caspase 3 (CC3) forms the core enzyme of apoptotic poly(ADP-ribose) polymerase (PARP) (29). CC3 mean intensity was used as a measure for CC3 expression. Across all experimental conditions, CC3 expression was higher in tumor cell aggregates (median 14.9; quartiles 11.8 to 22.0) than in stroma (10.2; 7.6 to 13.5; p<0.001).

**Figure 4:**
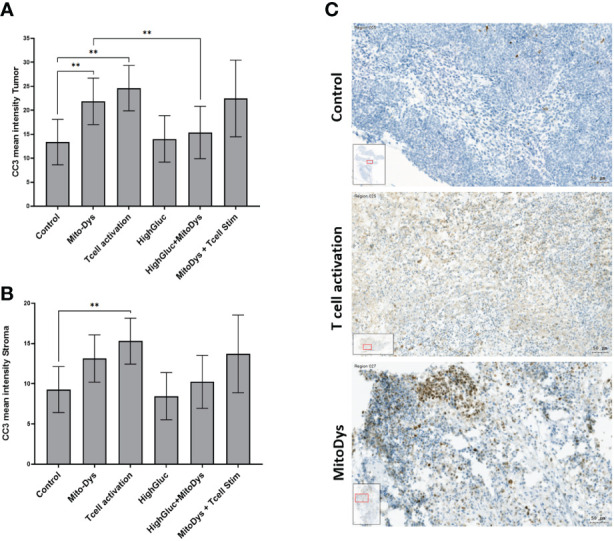
Mean intensity of CC3 in tumor **(A)** and stromal cells **(B)** of SC of HNSCC patients and images of CC3 staining of HNSCC SC **(C)**. **(A)** The mean CC3 intensity in tumor cell aggregates increased with MitoDys. CC3 expression was significantly lower with MitoDys+ HighGluc than with MitoDys alone, suggesting an mitigating effect of high glucose on MitoDys-induced CC3 expression. In addition, T cell activation increased CC3 expression in tumor cells, but this was not attenuated by concomitant MitoDys. **(B)** The mean CC3 intensities in the stroma behaved similar to the tumor cell aggregates but at a significantly lower level and the difference between control and MitoDys was not significant. The data is based on an evaluation of the whole slides (** significant < 0.01; error bars represent 95% CI). **(C)** CC3 staining of HNSCC of oral cavity for control condition (male, 37 years old), T cell activation (male, 37 years old) and MitoDys condition (male, 45 years old).CC3 positive cells increased in T cell stimulated SC. In MitoDys treated SC, especially in tumor cells CC3 positivity increased (bar: 50µm).

Looking at CC3 expression specifically in the tumor cell aggregates, MitoDys led to higher CC3 expression (21.9; 95% CI 17.6 to 27.2) than control conditions (13.4; 95% CI 11.5 to 15.6; p=0.003; **Figure 4A**). This effect was substantially mitigated by HighGluc in the medium (15.4; 95% CI 12.1 to 19.6). The difference of MitoDys + HighGluc compared to MitoDys alone was significant (p=0.03). HighGluc alone did not lead to any change in CC3 expression.

Similarly, T-cell activation led to an increase in CC3 expression to 24.6 (95% CI 18.0 to 33.6) when compared with control conditions (13.4; 95% CI 8.0 to 18.1; p=0.005). This effect was maintained with concomitant MitoDys (22.5; 95% CI 16.4 to 30.9; p vs. T-cell activation alone 0.69). In the tumor stroma, the results were essentially equivalent with significantly lower CC3 expression (**Figure 4B**). However, the difference of CC3 IHC-expression in control (9.3; 95% CI 6.4 to 12.1) and MitoDys (13.1; 95% CI 10.2 to 16.1) was not significant (p=0.065). Overall, T cell activation increased CC3 in both tumor aggregates and stroma. MitoDys however lead to increased CC3 in tumor cell aggregates, but not in stroma.

### 3.3 Granzyme B and CD8/KI67 co-expression

Together with perforin and granulolysin, the serine protease granzyme B forms the essential components of the cytotoxic pathway of lymphocytes and NK cells (30). Here, granzyme B expression was analyzed by immunohistochemistry and granzyme B release was quantified by dot blots of the supernatants under the 6 experimental conditions. In addition, we investigated the co-expression of CD8 and the proliferation marker KI67 in immunofluorescence using some slice cultures as examples.

**Figure 5:**
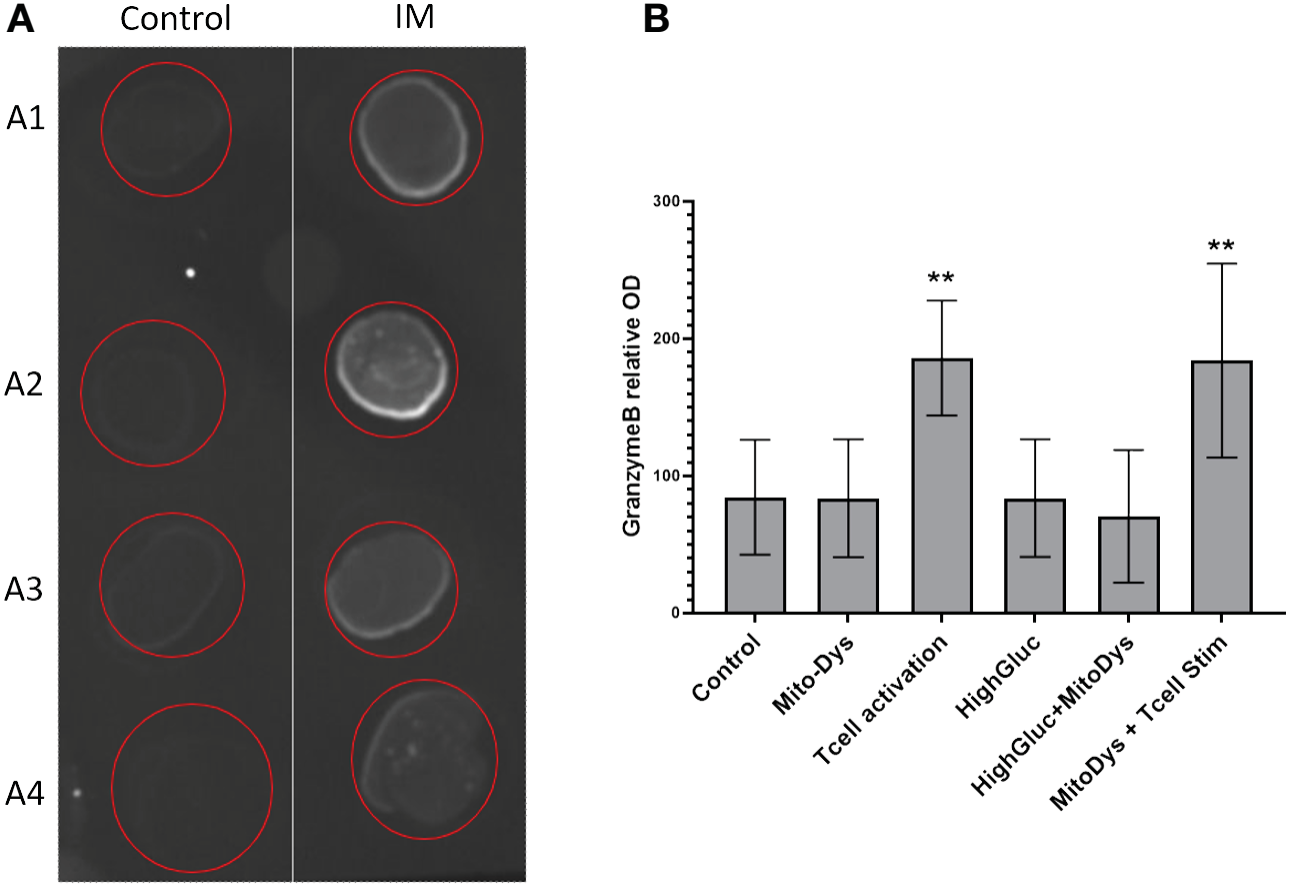
Granzyme B in culture supernatants. **(A)** Example of dot blot analysis of 4 different supernatants (A1-A4) for control conditions (Control) and T-cell stimulation (T cell activation); **(B)** Bar chart of mean optical densities (OD) per experimental condition, error bars correspond to 95% CI, **significant <0.01).

**Figure 6:**
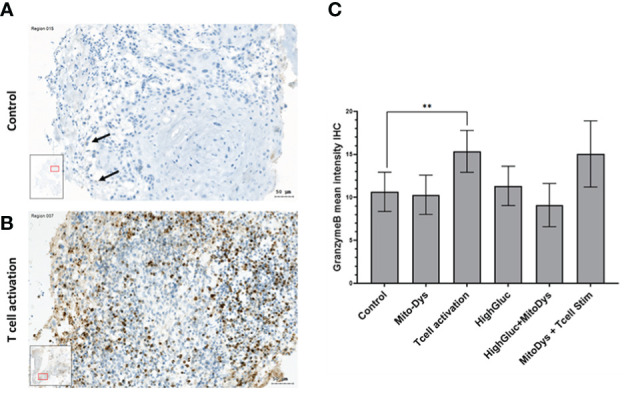
**(A)** Granzyme B-stained SC of an HNSCC of the oral cavity (male, 37 years) on day 3 under control conditions; only few Granzyme B reaction products in smaller inflammatory cells (arrows). **(B)** Under T-cell stimulation: numerous positive cells with granular reaction products of Granzyme (B) **(C)** Mean Granzyme B IHC intensities in SC of HNSCC per experimental condition. The data is based on an evaluation of the whole slide and not just the image section shown (** significant < 0.01; error bars represent 95% CI; inlets: position of the image section in the whole slide).

Granzyme B concentration in the supernatant increased following T cell stim (p=0.005) and this effect was not counteracted by simultaneous induction of MitoDys (p vs. T cell stim alone =0.54; **Figure 5**). In detail, T cell activation resulted in a higher relative granzyme B optical density in the dot blots (189.8; 95% CI 148.2 to 243.1) than the control conditions (93.9; 95% CI 62.3 to 141.5; p=0.005), as did T cell activation in combination with MitoDys (214.7; 95% CI 159.5 to 289.1; p<0.001). Similarly, compared to control (10.7; 95% CI 8.7 to 13.1), mean granzyme B intensity increased following T cell stim (15.4; 95% CI 12.1 to 19.4; p=0.015; **Figure 6**). This effect was not altered by additional induction of MitoDys (15.1; 95% CI 12.5 to 18.1; p vs. T cell stim alone 0.9). Thus, MitoDys did not lead to a dysfunction of the tumor infiltrating lymphocytes of the slice cultures.

**Figure 7:**
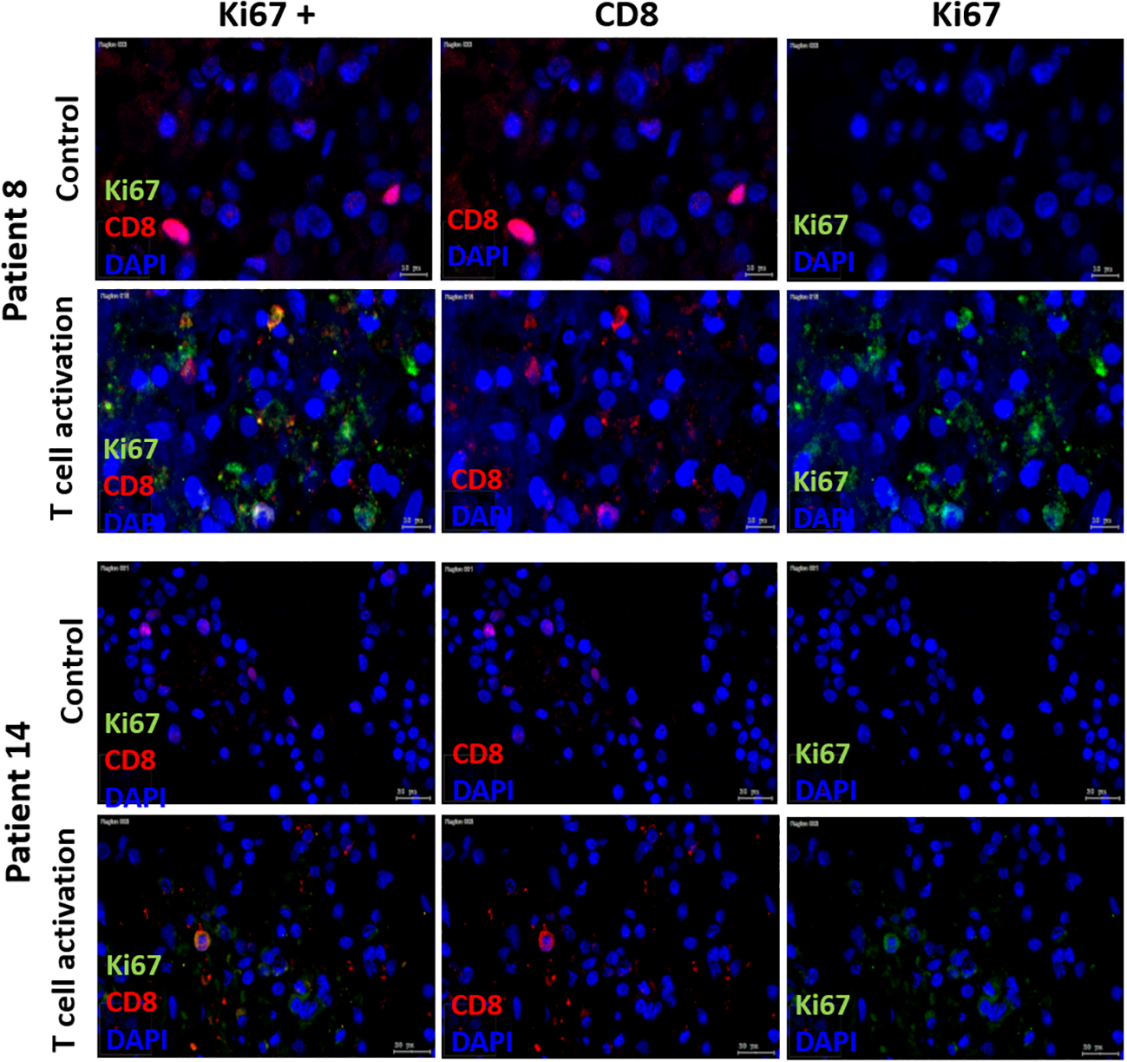
Immune fluorescent Ki67/CD8, CD8 and Ki67 staining. Double-staining with Ki67/CD8 of SC after day 3 in two patients (Patient 8 = HNSCC oropharynx, female, 66 years old; Patient 14= HNSCC oral cavity, male, 57 years old) in control and T cell stim conditions. In both patients, Ki67 staining alone in control condition showed no positive Ki67 cells. With T cell stim, amount of Ki67 pos. cells increased.

According to the manufacturer’s data sheet, T cell expansion requires repeated stimulation with rIL2 and cultivation for more than seven days, which we did not do in this study. Nevertheless, we used some slice cultures to test whether T cell proliferation can also be observed after activation with the T cell activation and expansion kit. For this purpose, the co-expression of CD8 and KI67 was examined using fluorescence microscopy in some of the SC. Double positivity was investigated in chosen sections and different frequencies of double positive reactions were found, due to unspecific immunofluorescence stainings, regardless of computerized quantitative analysis of double reactivity. Therefore, co-expression could be detected in some, but not all SC (**Figure 7**).

### 3.4 PD-1 expression

**Figure 8:**
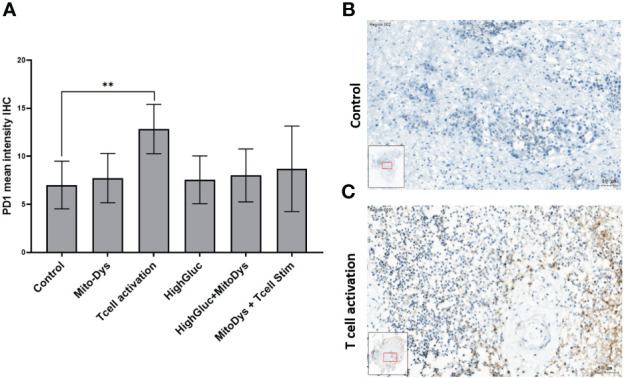
Mean PD-1 intensity in IHC of SC from HNSCC patients after 3 days of cultivation under 6 different culture conditions **(A)**. Compared to control, T cell stimulation increased the mean PD-1 intensity (p=0.004; ** significant < 0.01; error bars represent 95% CI). **(B)** PD-1 IHC staining of a SC of an HNSCC of the oral cavity (male, 37 years old) under control conditions. No PD-1 reaction products in the larger tumor cells or the smaller inflammatory cells in the tumor stroma **(C)** PD-1 IHC of the SC under T-cell stimulation. Positive cells show a ring-shaped membrane reaction of PD1.

The IHC expression of PD-1 was used as an indicator for possible T-cell exhaustion. Compared to control, MitoDys alone did not increase PD-1 expression (p=0.43). A higher mean PD-1 expression compared to the control (7.0; 95% CI 5.2 to 9.5) was only found after T-cell stimulation (12.5; 95% CI 9.3 to 16.9; p=0.004). In particular, there was no increase in PD-1 expression with concomitant induction of MitoDys (p=0.08, **Figure 8**).

## 4 Discussion

The current understanding of the pathogenesis, progression, and therapy of HNSCC has reached a high level of complexity. Sequencing of the tumor genome has increasingly improved the identification of multiple mutations in tumor cells (31). Clinically relevant progress has been made in understanding the complex mechanisms of immune evasion of tumor cells (9). However, it is becoming more evident that the complex interactions of tumor and immune cells with other mutually influencing elements of the TME have a significant impact on disease progression (32). Moreover, the hostile metabolic conditions in the TME are becoming a center of interest as they may foster tumor progression and interfere with antitumor immune response (12, 33). Current knowledge on the impact of these conditions on lymphocyte antitumor response is based on studies in PBMC derived lymphocytes or lymphocyte cell lines (34). Recent studies using these models report that mitochondria play a critical role in T cell metabolism, differentiation, and signaling (11). During activation, T cells undergo metabolic reprogramming, shifting from an OXPHOS-dominated metabolism in naïve CD8+ T cells to a more glycolysis-dominated metabolism in CD8+ effector T cells (35). Lymphocyte cell lines are heterogeneous, particularly in terms of glycolytic or aerobic oxidative activity (36). However, the extent to which these *in vitro* data apply to tumor infiltrating lymphocytes is unknown. The most direct method to study immune activity of tumor-infiltrating lymphocytes (TILs) is to examine them in their control environment. Cultures of patient derived tumor tissue slices provide insights into the complex interactions within their TME, reflect their high inter- and intra-individual variability and may maintain the metabolic conditions ex vivo (37). The tumor SC of patients with HNSCC remained well preserved over the experimental period of 3 days, and the cell density on day 3, at over 9000 cells/mm², was markedly higher than in a previous study (21). This may be due to the gentle cutting technique of the tumor biopsies with the Compresstome® (38).

### 4.1 Tumor infiltrating T cells retain their cytotoxic activity in the hostile TME of HNSCC

However, the decrease in cell density compared to post-biopsy and the high proportion of tumor cells expressing the apoptosis marker CC3 (median 44%; quartiles 28% to 71%) under control conditions indicate that the hostile metabolic conditions were preserved within the TME of SC. Despite this, TILs remained responsive to immune stimulation. Stimulation with a commercial T cell activation kit led to increased granzyme B expression in tumor cells, heightened granzyme B release into culture supernatants (**Figure 4**), and upregulated pro-apoptotic cleaved caspase 3 in tumor cells under control conditions (**Figure 3**), aligning with previous publications (22). The data sheet of the T cell activation kit indicates that T cell expansion can be expected after 2 additional stimulations with rIL2, 7 days apart. Nevertheless, we observed co-expression of CD8 and the proliferation marker KI67 in some SC after only 3 days. This was not the case in controls (**Figure 6**), suggesting that the capacity for T cell expansion was also maintained in TILs of HNSCC.

### 4.2 Tumor infiltrating T cells remain responsive after additional induction of mitochondrial dysfunction

To induce MitoDys in the TME of SC, we added FFCP/oligomycin A to force the cells into glycolysis. A combination of FFCP/oligomycin A inhibits OXPHOS and increases glycolysis in HNSCC tumor cells (17) and lymphocytes (39). Our observations confirmed that FFCP/oligomycin A increased apoptosis makers in HNSCC tumor cells, signifying effective MitoDys induction within the TME of HNSCC slice cultures. This effect was attenuated by high glucose concentration in the medium, indicating that glycolysis was effectively stimulated and that tumor cells used extracellular glucose to evade apoptosis under these glycolytic conditions. While FFCP/oligomycin A induced apoptosis in tumor cells, it had no effect on cytotoxic T cell effector function. The granzyme B response and tumor cell apoptosis under FCCP/oligomycin A conditions was comparable to that with T cell stimulation alone (**Figures 3**–**5**).

In Raji cells, a high glucose concentration in lymphocyte cultures under glycolytic conditions increased the cytotoxic T-cell response through mechanisms that are not yet fully understood (34). However, increasing glucose concentrations from 5.8 mmol/l to 25 mmol/l caused neither an increase in the granzyme B response nor an increase in the expression of the apoptosis marker CC3 in tumor cells (**Figures 3**, **4**). *In vivo*, pro-cytotoxic effects of T cells may not increase via high glucose concentration, as it has been demonstrated in studies on mice (40). Furthermore, tumor cell apoptosis may not be affected by high glucose, due to its positive effects on tumor cells (41). The available tumor tissue was not sufficient to investigate the effect of a high glucose concentration on T-cell activation under FCCP/oligomycin A stress.

Using PD-1 expression, we investigated possible effects on T cell exhaustion, which is discussed as a possible cause for the failure of ICI therapy. We assume that PD-1 is only expressed on immune cells and not on HNSCC tumor cells. T cell activation increased PD-1 expression. Assuming that TILs in SC are constantly under metabolic stress, this supports reports that T cell stimulation under metabolic stress promotes T cell exhaustion (42). Interestingly, the upregulation of PD-1 may have been attenuated by FCCP/oligomycin A (**Figure 6**; p vs. T cell activation alone: 0.078), suggesting that PD-1 upregulation could be an OXPHOS-dependent process (10). There are several examples of persistently OXPHOS-dependent processes in lymphocytes that have largely switched to glycolysis after activation.

### 4.3 Limitations and perspectives

During the cultivation, staining and cutting of the SC, some samples were lost. Consequently, in some culture conditions, only 94 SC remained for analysis instead of the initially planned 101 SC. Co-expression of PD-1 and TIM-3 would have been a more specific indicator of T cell exhaustion than PD-1 expression alone (43). In this study, FCCP and oligomycin A significantly increased tumor cell apoptosis in HNSCC without affecting cytotoxic T cell function. A clinically suitable substitute for FCCP and oligomycin is IACS-010759 [48], is currently in clinical trials. This inhibitor blocks complex I in the mitochondria and could also have antitumor effects. Furthermore, SC are highly heterogeneous: Even within one individual, slice composition varies greatly.

## 5 Conclusions

Tumor infiltrating lymphocytes in SC of HNSCC could be activated and led to apoptosis of tumor cells. OXPHOS played a minimal role in the activation of TILs in SC of HNSCC. In contrast to some studies on cultured lymphocytes, a higher glucose concentration did not increase the cytotoxic T cell activity of TILs in HNSCC slice cultures. As in a previous study, slice cultures were found to be a useful model for the investigation of immune processes in the TME of HNSCC.

## Data availability statement

The raw data supporting the conclusions of this article will be made available by the authors, without undue reservation.

## Ethics statement

The studies involving humans were approved by Ethics Committee of the Medical University of Innsbruck. The studies were conducted in accordance with the local legislation and institutional requirements. The participants provided their written informed consent to participate in this study. Written informed consent was obtained from the individual(s) for the publication of any potentially identifiable images or data included in this article.

## Author contributions

MG: Conceptualization, Data curation, Formal Analysis, Investigation, Methodology, Project administration, Writing – original draft, Writing – review & editing, Visualization. AnR: Conceptualization, Data curation, Formal Analysis, Funding acquisition, Investigation, Methodology, Project administration, Writing – original draft, Writing – review & editing. JD: Conceptualization, Data curation, Formal Analysis, Investigation, Methodology, Project administration, Resources, Supervision, Validation, Writing – original draft, Writing – review & editing. RH: Data curation, Formal Analysis, Writing – original draft, Writing – review & editing, Methodology. MS: Data curation, Formal Analysis, Writing – original draft, Writing – review & editing, Methodology. DD: Data curation, Formal Analysis, Writing – original draft, Writing – review & editing. TS: Conceptualization, Data curation, Formal Analysis, Writing – original draft, Writing – review & editing. JF: Conceptualization, Data curation, Formal Analysis, Writing – original draft, Writing – review & editing, Resources. CS: Data curation, Formal Analysis, Methodology, Resources, Writing – original draft, Writing – review & editing. MK: Writing – original draft, Writing – review & editing, Supervision, Conceptualization. SuS: Investigation, Visualization, Writing – original draft, Writing – review & editing, Conceptualization, Methodology. SS: Writing – original draft, Writing – review & editing, Conceptualization. AvR: Writing – original draft, Writing – review & editing, Formal Analysis, Validation. MM: Conceptualization, Formal Analysis, Methodology, Resources, Writing – original draft, Writing – review & editing. BH: Conceptualization, Resources, Supervision, Writing – original draft, Writing – review & editing. HR: Conceptualization, Formal Analysis, Funding acquisition, Methodology, Supervision, Validation, Writing – original draft, Writing – review & editing, Data curation.
