# Supplementary material for: Cytotoxic response of tumor-infiltrating lymphocytes of head and neck cancer slice cultures under mitochondrial dysfunction
Source: Front Oncol. 2024 Mar 7;14:1364577. doi: 10.3389/fonc.2024.1364577 (PMC10954813; doi:10.3389/fonc.2024.1364577)

**Supplementary Data**

Supplement Tables 1-6: Original segmentation settings and parameter for cell nuclei and cytoplasmatic stainings (Granzyme B, PD1 and Cleaved Caspase 3) in HistoQuest.

Supplementary Table 1: Nuclear segmentation Hematoxylin for Granzyme B

| **Nuclear segmentation Hematoxylin for Granzyme B** |  |
| --- | --- |
| Color | blue |
| Intensity | 227 |
| Mixture Percent | 90 |
| Nuclei Size | 29 |
| Remove small-sized objects | 1 |
| Remove weakly stained objects | 1 |
| Automatic Background Threshold | No |
| Background Threshold | 16 |
| Virtual Channel | nuclear |
| Post Processing Order | Remove, Merge |
| Remove Labels | No |
| Use Merging Rules | No |

Supplementary Table 2: IHC segmentation Granzyme B

| **IHC segmentation Granzyme B** |  |
| --- | --- |
| Color | brown |
| Intensity | 134 |
| Use Ring Mask | Yes |
| Uinterior Radius | -0,14 µm |
| Exterior Radius | 0,28 µm |
| Use Identified Cell Mask | Outside&Inside |
| Max Growing Steps | 0,56 µm |
| Skip Steps | 0 µm |
| Use Nuclei Mask | No |
| Automatic Background Threshold | No |
| Background Threshold | 6 |

Supplementary Table 3: Nuclear segmentation Hematoxylin for PD1

| **Nuclear segmentation Hematoxylin for PD1** |  |
| --- | --- |
| Color | blue |
| Intensity | 255 |
| Mixture Percent | 48,5 |
| Nuclei Size | 29 |
| Remove small-sized objects | 1 |
| Remove weakly stained objects | 1 |
| Automatic Background Threshold | No |
| Background Threshold | 9 |
| Virtual Channel | nuclear |
| Post Processing Order | Remove, Merge |
| Remove Labels | No |
| Use Merging Rules | No |

Supplementary Table 4: IHC segmentation PD1

| **IHC segmentation PD1** |  |
| --- | --- |
| Color | brown |
| Intensity | 134 |
| Use Ring Mask | Yes |
| Uinterior Radius | -0,14 µm |
| Exterior Radius | 0,28 µm |
| Use Identified Cell Mask | Outside&Inside |
| Max Growing Steps | 0,56 µm |
| Skip Steps | 0 µm |
| Use Nuclei Mask | No |
| Automatic Background Threshold | No |
| Background Threshold | 6 |

Supplementary Table 5: Nuclear segmentation Hematoxylin for Cleaved Caspase 3

| **Nuclear segmentation Hematoxylin for Cleaved Caspase 3** |  |
| --- | --- |
| Color | blue |
| Intensity | 227 |
| Mixture Percent | 90 |
| Nuclei Size | 44 |
| Remove small-sized objects | 1 |
| Remove weakly stained objects | 1 |
| Automatic Background Threshold | No |
| Background Threshold | 16 |
| Virtual Channel | nuclear |
| Post Processing Order | Remove, Merge |
| Remove Labels | No |
| Use Merging Rules | No |

Supplementary Table 6: IHC segmentation Cleaved Caspase 3

| **IHC segmentation Cleaved Caspase 3** |  |
| --- | --- |
| Color | brown |
| Intensity | 134 |
| Use Ring Mask | Yes |
| Uinterior Radius | 5,58 µm |
| Exterior Radius | 3,07 µm |
| Use Identified Cell Mask | Outside&Inside |
| Max Growing Steps | 0,56 µm |
| Skip Steps | 0 µm |
| Use Nuclei Mask | Yes |
| Automatic Background Threshold | Yes |
| Background Threshold | [5,255] |

Supplementary Figure 1: Stratification between tumor and stroma cells using hematoxylin equivalent diameter and hematoxylin area. Using the above detailed parameters, cell nuclei were segmented and cytoplasmatic cleaved caspase 3 (CC3) reactions were optimally recognized. HistoQuest software offers morphological parameters for distinguishing among different cell types. Among these parameters, hematoxylin equivalent diameter and hematoxylin area, achieved sufficient differentiation between tumor and stroma cells. From the scatter gram, the lower left quadrate was further used as input gate for stroma cells and the upper right quadrate as tumor cells. In the next step CC3, mean intensity was separately determined in the cells belonging to these input gates.


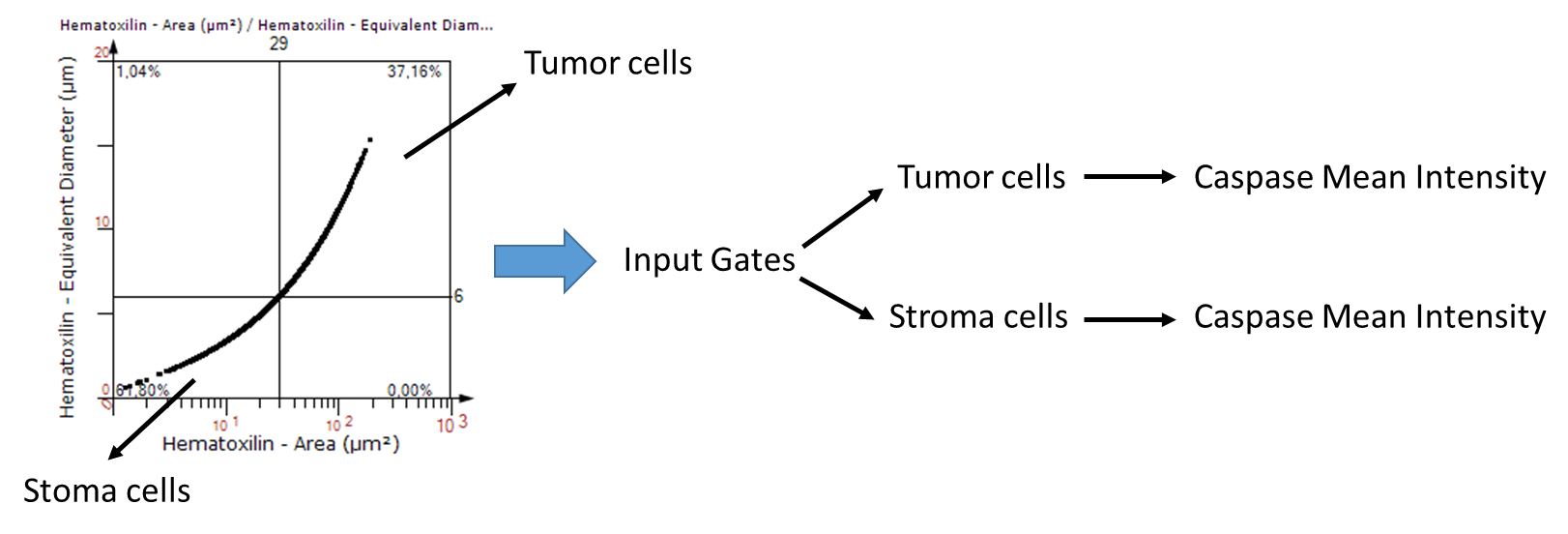

Supplement: Supplementary file 1 [file DataSheet_1.docx]
